# Use of qualitative research in World Health Organisation guidelines: a document analysis

**DOI:** 10.1186/s12961-024-01120-y

**Published:** 2024-04-04

**Authors:** Melissa Taylor, Paul Garner, Sandy Oliver, Nicola Desmond

**Affiliations:** 1 Department of Clinical Sciences, Liverpool School of Tropical Medicine, Liverpool, UK; 2 EPPI-Centre, Social Science Research Unit, UCL Institute of Education, University College London, London, UK; 3 Africa Centre for Evidence, Faculty of Humanities, University of Johannesburg, Johannesburg, South Africa; 4 Department of International Public Health, Liverpool School of Tropical Medicine, Liverpool, UK

**Keywords:** Qualitative evidence synthesis, Qualitative research, Guideline development

## Abstract

**Background:**

Guidelines depend on effect estimates, usually derived from randomised controlled trials, to inform their decisions. Qualitative research evidence may improve decisions made but where in the process and the methods to do this have not been so clearly established. We sought to describe and appraise how qualitative research has been used to inform World Heath Organization guidance since 2020.

**Methods:**

We conducted a document analysis of WHO guidelines from 2020 to 2022. We purposely sampled guidelines on the topics of maternal and newborn health (MANH) and infectious diseases, as most of the qualitative synthesis to date has been conducted on these topics, likely representing the ‘best case’ scenario. We searched the in-built repository feature of the WHO website and used standardised search terms to identify qualitative reporting. Using deductive frameworks, we described how qualitative evidence was used to inform guidelines and appraised the standards of this use.

**Results:**

Of the 29 guidelines, over half used qualitative research to help guide decisions (18/29). A total of 8 of these used qualitative research to inform the guideline scope, all 18 to inform recommendations, and 1 to inform implementation considerations. All guidelines drew on qualitative evidence syntheses (QES), and five further supplemented this with primary qualitative research. Qualitative findings reported in guidelines were typically descriptive, identifying people’s perception of the benefits and harms of interventions or logistical barriers and facilitators to programme success. No guideline provided transparent reporting of how qualitative research was interpreted and weighed used alongside other evidence when informing decisions, and only one guideline reported the inclusion of qualitative methods experts on the panel. Only a few guidelines contextualised their recommendations by indicating which populations and settings qualitative findings could be applied.

**Conclusions:**

Qualitative research frequently informed WHO guideline decisions particularly in the field of MANH. However, the process often lacked transparency. We identified unmet potential in informing implementation considerations and contextualisation of the recommendations. Use in these areas needs further methods development.

**Supplementary Information:**

The online version contains supplementary material available at 10.1186/s12961-024-01120-y.

## Background

Evidence-informed guidance usually includes a critical summary of one or more systematic reviews of reliable research findings to inform the decisions. For simple clinical questions which assess the efficacy of a new drug, systematic reviews of randomised control trials may provide the most appropriate information [1]. Making recommendations about drugs, vaccines and public health interventions all require reflection on the acceptability or appropriateness of an intervention, and this requires different forms of evidence and types of research [2]. The value of qualitative methods lies in their ability to pursue systematically ‘what’, ‘why’ or ‘how’ questions that are not easily answerable by experimental methods [3].

There is an increasing recognition of the importance of the social determinants of health in policy making, given the complex nature of most public health issues [4]. Qualitative research methods are particularly adept to explore these findings from the individual, community or broader system level [4, 5]. Qualitative research may also range from descriptive to explanatory in nature [6]. Descriptive findings address people’s views or experiences, such as the perception of personal benefits and harms of interventions, and the trade-offs between these. Descriptive findings may also identify and describe unintended consequences of the proposed intervention. Finally, they may identify logistical barriers and facilitators to programme success [7, 8]. These aspects are particularly valuable, as they bring forth the patient and health worker voice in decision making [9].

Explanatory findings, on the other hand, link descriptive perspectives or experiences to aspects of psychological, historical, cultural, economic, social, environmental and political context [6]. In doing so, they help generate a theoretical understanding of ‘why’ perceptions and experiences occur and may have broader applications to related contexts [6]. Here qualitative findings may be used to explain how personal attributes and lifestyle impact individuals, how local context impacts group choice to access treatment or diagnosis or how broad structural and health systems can impede their ability to access, benefit or trust health interventions [7, 8].

Guideline developers such as the World Health Organisation (WHO) are beginning to draw on qualitative research to inform their decisions [2], aided by the methodological developments of systematic reviews of qualitative research, known as qualitative evidence syntheses (QES) and their appraisal [10]. Previous research has documented examples on how qualitative research has so far informed guideline processes, including identifying relevant outcomes, evaluating evidence to produce recommendations and developing implementation considerations [11–13]. However, it remains unclear how often qualitative research is actually used for these purposes. Further, it is thought that qualitative research does not always fit well within the ‘summary-based and compartmentalised structure’ of the guideline framework [12], given the wide range of aims of qualitative research, from describing people’s views to explaining the impact of structural barriers to treatment access. Documenting which of these the WHO has drawn on so far will help to further refine guidance for the uptake of qualitative research by Identifying areas of unmet potential.

Furthermore, as with any guideline development, those preparing the reviews and the panels using them need to provide transparent reporting and rigorous appraisal akin to those historically practised with quantitative research in decision-making [12, 14]. However, so far, no methodological guidance exists on how best to systematically draw on and evaluate qualitative findings during guideline processes [9], and it is unclear how often these standards are achieved [11–13].

Our aim is to describe how qualitative evidence has been used in existing WHO guideline development procedures and appraise the standards of this inclusion.

## Methods

We used a study design of document analysis to systematically describe and appraise WHO guidelines. Document analysis is a qualitative method commonly used in health policy analysis [15], which aims to synthesise and appraise textual data to elicit meaning, gain understanding and develop empirical knowledge [16]. This necessitates a systematic approach; however, standardised methodologies are lacking [15]. To ensure rigour, we drew on Kayesa and Shung-King [15], who identified the key steps reported in document analyses: adopting clear inclusion criteria for documents and clear procedures for identifying documents, coding them and extracting data; applying a clear analytical framework to analyse the role of qualitative research cited in policy documents; and presenting the findings of each stage of the process from searching for documents to answering the research question.

### Guideline retrieval

A scoping search of the Cochrane Library [17] identified that QES were most frequently conducted on MANH (6/23 QES) and infectious disease topics (7/23 QES). For this reason, we chose to focus our analysis on these topic areas, as whilst not exhaustive, they may represent the ‘best case scenario’.

We used the in-built document repository feature on WHO’s website [18] to identify guideline documents. Therefore, only documents published on this web page were eligible for inclusion. Grey literature was not included. The web page allowed for filtering by publication type and year, which was restricted to ‘guideline’ in 2020 and 2021. A 2-year period was chosen to reflect the most current practices of qualitative research at the time of the search. The lead author (M.T.) then screened the guideline titles in the search results for topics relating to Maternal and Newborn Health (MANH) and infectious disease. MANH was defined as any topic covering the health of women during pregnancy, childbirth and the post-partum period and babies’ first month of life. Infectious disease was defined as any topic covering the prevention, diagnosis and treatment of all diseases acquired through human–human or animal–human transmission, including vector-borne diseases. A table detailing the excluded guidelines and justification for this exclusion can be found in Additional file 1: Excluded studies. The final list of included and excluded guidelines was approved by the entire author team.

The unit of analysis used in this study was the section of text describing a qualitative finding within a guideline document. As a result, we performed a second search within the included guideline documents to identify any qualitative reporting. We defined a qualitative study as one that collected data using qualitative methods such as ethnographic observations, in‐depth interviews, focus group discussions and open‐ended survey questions. Appropriate analysis methods included, for example, thematic analysis, narrative analysis, framework analysis, and grounded theory. While we acknowledge that mixed methods studies may contribute qualitative findings, for the purpose of this study they were excluded, as it was not possible to identify which findings had been derived from quantitative or qualitative methods. Initial reading of a sample of three guidelines in-depth identified terms that accompanied qualitative reporting. We then performed a key-word search for the following terms in all guidelines to identify qualitative reporting: ‘qualitative’, ‘accept*’, ‘value’, ‘equit*’, ‘feasib’, ‘interview’ or ‘focus-group discussion’. Sections of text containing the keywords were checked against their corresponding citation to ensure the findings were derived from qualitative studies.

### Data extraction and analysis

**Fig. 1:**
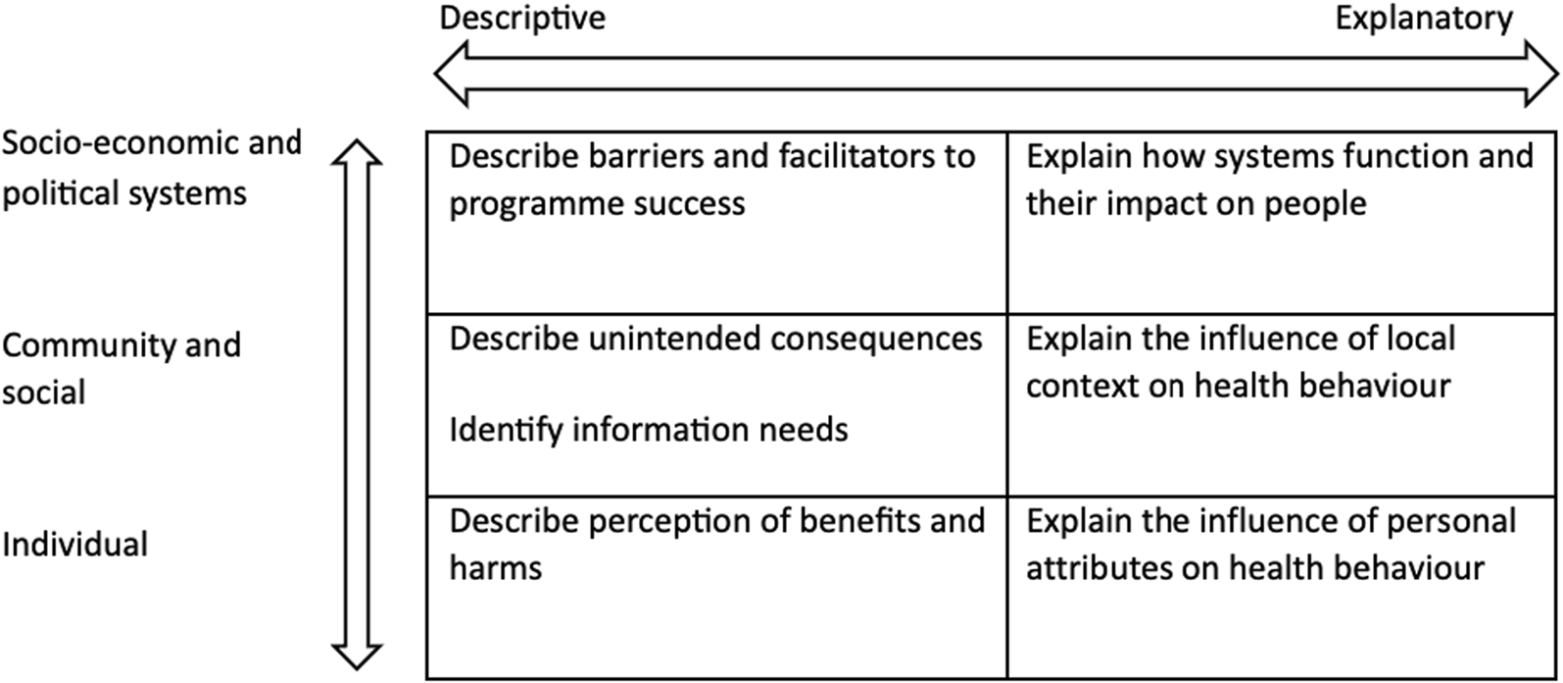
Matrix of how qualitative research can contribute to guideline development

Data analysis occurred in three phases. First, given the broad range of potential qualitative findings, we sought to understand what ‘type’ guidelines typically drew on. To achieve this, we developed a deductive framework informed by the literature. We crossed (1) the nested individual, community and broader system ecosystems within social determinants of health theory against (2) descriptive to explanatory qualitative research methodology. Within this, we populated the matrix with qualitative research aims derived from literature and discussed in the background of this paper. This provided us with a theoretical overview of the potential contribution of qualitative research (Fig. 1). We then coded each qualitative finding contained within guidelines with one of these aims. The framework was validated on a selection of guidelines, which led to the inclusion of one inductive aim of qualitative research: to understand information needs.

We next sought to describe how qualitative research was identified by the guideline and how it was used to inform the scope of the guideline, the intervention recommendation and implementation considerations. Finally, we sought to appraise how qualitative research had been used using analogous standards expected and practised for quantitative methods in decision-making processes. Table 1 guided this process. Extraction domains and questions were initially identified a priori, and any new questions that arose during analysis were shared and discussed within the team to ensure they were appropriate. If new questions were added to the data extraction tool (Table 1), all guidelines were re-analysed to ensure a complete dataset.

**Table 1:** Domains used to guide data extraction

Domain	Questions
How qualitative research was used	
Retrieval	How was qualitative research identified or sought for inclusion in the guidelines? What qualitative study designs were included? What role does qualitative research provide here?
Informing scope	How many guidelines used qualitative research to inform their scope? What was the nature of their inclusion? What role does qualitative research provide here?
Informing recommendations	How many guidelines used qualitative research to inform their recommendation, including the domains of feasibility, acceptability, values and preferences and equity? How often did guidelines provide a rationale for their judgement of qualitative research? What role does qualitative research provide here?
Informing implementation considerations	How many guidelines used qualitative research to inform their implementation considerations? What was the nature of their inclusion?
Standards of qualitative research use	
Certainty	Did guidelines report the certainty of evidence alongside QES findings or report a quality appraisal of stand-alone primary studies?
Transparency	Is it clear how the qualitative research supported the decision? Is it clear how this research was discussed and evaluated? How was consensus achieved?
Specificity	Is there any discussion of the populations these findings are relevant to. Has it been generalised to the point where it may not be useful any more?
Reflexivity	What qualitative skills did the panel have and have they described who was involved in summarising qualitative research?

## Results

### Search results

**Fig. 2:**
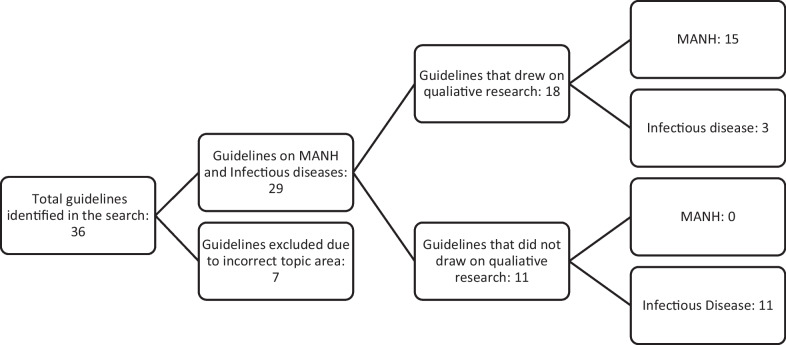
Overview of guideline search process

Between 2020 and 2022, the WHO published 29 guidelines on the topics MANH and infectious diseases. Seven guidelines were excluded as they did not cover the chosen topics areas. Of the 29 included guidelines, 18 (62%) incorporated qualitative research to inform either the scope, recommendation or implementation considerations. Of the 18 guidelines that used qualitative research, 15/18 guidelines were on topics of MANH, in contrast to 3/18 on infectious diseases. An overview of the search results is shown in Fig. 2 below, and a summary of all included guidelines is detailed in Table 2.

**Table 2:** A summary of all guidelines identified in the search and their use of qualitative evidence

Guideline	Year	Retrieval process	Type of qualitative research (n=)	Quality appraisal by guideline	Did qualitative research inform the question?	Did qualitative research inform feasibility?	Did qualitative research inform acceptability?	Did qualitative research inform values and preferences?	Did qualitative research inform equity?	Did qualitative research inform the implementation considerations	Total number of domains that draw on qualitative research
Infectious diseases guidelines											
Screening and treatment of cervical pre-cancer lesions for cervical cancer prevention, second edition: use of mRNA tests for human papillomavirus (HPV) [28]	2021	Commissioned	QES (2)	None for QES	Yes	No	Yes	No	No	No	2
HIV prevention, testing, treatment, service delivery and monitoring [27]	2021	Unclear	Primary study (26) and QES (7)	None for primary studies or QES	Yes	No	Yes; 9/99 recommendations	Yes; 1/99 recommendations	Yes; 1/99 recommendations	Yes	4
Management of symptomatic sexually transmitted infections [45]	2021	N/A	N/A	N/A	No	No	No	No	No	No	0
Preventive chemotherapy for the control of *Taenia solium* taeniasis [46]	2021	N/A	N/A	N/A	No	No	No	No	No	No	0
Hepatitis C virus self-testing [47]	2021	N/A	N/A	N/A	No	No	No	No	Yes	No	0
Plague management: revised recommendations for the use of rapid diagnostic tests, fluoroquinolones for case management and personal protective equipment for prevention of post-mortem transmission [48]	2021	N/A	N/A	N/A	No	No	No	No	No	No	0
Infant feeding in areas of Zika virus transmission [31]	2021	Commissioned	QES (2)	Quality appraisal for studies included in QES	No	Yes	No	Yes	No	No	2
Antigen-detection in the diagnosis of SARS-CoV-2 infection: interim guidance	2021	N/A	N/A	N/A	No	No	No	No	No	No	0
Mass drug administration of azithromycin to children under 5 years of age to promote childhood survival [49]	2020	N/A	N/A	N/A	No	No	No	No	No	No	0
Prevention of sexual transmission of Zika virus [50]	2020	N/A	N/A	N/A	No	No	No	No	No	No	0
Consolidated HIV strategic information guidelines: driving impact through programme monitoring and management [51]	2020	N/A	N/A	N/A	No	No	No	No	No	No	0
Management of pregnant and breastfeeding women in the context of Ebola virus disease [52]	2020	N/A	N/A	N/A	No	No	No	No	No	No	0
Diagnosing and managing disseminated histoplasmosis among people living with HIV [53]	2020	N/A	N/A	N/A	No	No	No	No	No	No	0
Maternal and newborn health guidelines											
Antiplatelet agents for the prevention of preeclampsia [20]	2021	Searched	QES (1)	GRADE CERQual	No	Yes	Yes	Yes	No	No	3
Antenatal care recommendations for a positive pregnancy experience. Nutrition interventions update: Zinc supplementation [21]	2021	Unclear	QES (2)	GRADE CERQual	Yes	Yes	Yes	Yes	No	No	4
Vaginal preparation with antiseptic agents for women undergoing caesarean section [30]	2021	Commissioned	QES (2)	GRADE CERQual	No	No	No	Yes	No	No	1
Choice of antiseptic agent and method of application for preoperative skin preparation for caesarean section [54]	2021	Unclear	QES (1) Primary (3)	GRADE CERQual for QES but no quality appraisal for primary	No	No	No	Yes	No	No	1
Routine antibiotic prophylaxis for women undergoing operative vaginal birth [55]	2021	Unclear	QES (1)	GRADE CERQual for QES	No	No	No	Yes	No	No	1
Prophylactic antibiotics for women undergoing caesarean section [56]	2021	Unclear	QES (1) Primary (1)	GRADE CERQual for QES but no quality appraisal for primary	No	No	No	Yes	No	No	1
Uterine balloon tamponade for the treatment of postpartum haemorrhage [32]	2021	Commissioned	QES (2)	GRADE CERQual for QES but no quality appraisal for primary	No	Yes	Yes	Yes	No	No	3
Maternal and newborn care for a positive postnatal experience [25]	2020	Commissioned	QES (12); Primary study (7)	GRADE CERQual for QES and none for primary studies	Yes	Yes	Yes	Yes	No	No	4
Umbilical vein injection of oxytocin for the treatment of retained placenta [29]	2020	Commissioned	QES (2)	GRADE CERQual	No	Yes	Yes	Yes	Yes	No	4
Drug treatment for non-severe hypertension in pregnancy [22]	2020	Commissioned	QES (2) Primary study (1)	GRADE CERQual for one QES and none for primary study	Yes	Yes	Yes	Yes	No	No	4
Antenatal care recommendations for a positive pregnancy experience. Nutrition interventions update: micronutrient supplementation [19]	2020	Searched	QES (1)	GRADE CERQual	Yes	Yes	Yes	Yes	No	No	4
Calcium supplementation during pregnancy for prevention of preeclampsia and its complications [24]	2020	Unclear	QES (3); primary study (1)	GRADE CERQual for all QES and none for primary study	No	Yes	Yes	Yes	No	No	3
Antenatal care recommendations for a positive pregnancy experience. Nutrition interventions update: vitamin D supplements during pregnancy [23]	2020	Unclear	QES (1)	GRADE CERQual	No	Yes	Yes	Yes	No	No	3
Advance misoprostol distribution to pregnant women for prevention of postpartum haemorrhage [33]	2020	Search	QES (3)	GRADE CERQual	Yes	Yes	Yes	Yes	Yes	No	4
Routes of oxytocin administration for the prevention of postpartum haemorrhage after vaginal birth [34]	2020	Search	QES (2)	GRADE CERQual	Yes	Yes	Yes	Yes	Yes	No	5

Below follows a narrative summary of where the 18 guidelines used qualitative evidence in informing their scope, decisions and implementation considerations. This is followed by an appraisal of this use according to the pre-specified domains of transparency, specificity, certainty and reflexivity.

### How qualitative research was used

**Fig. 3:**
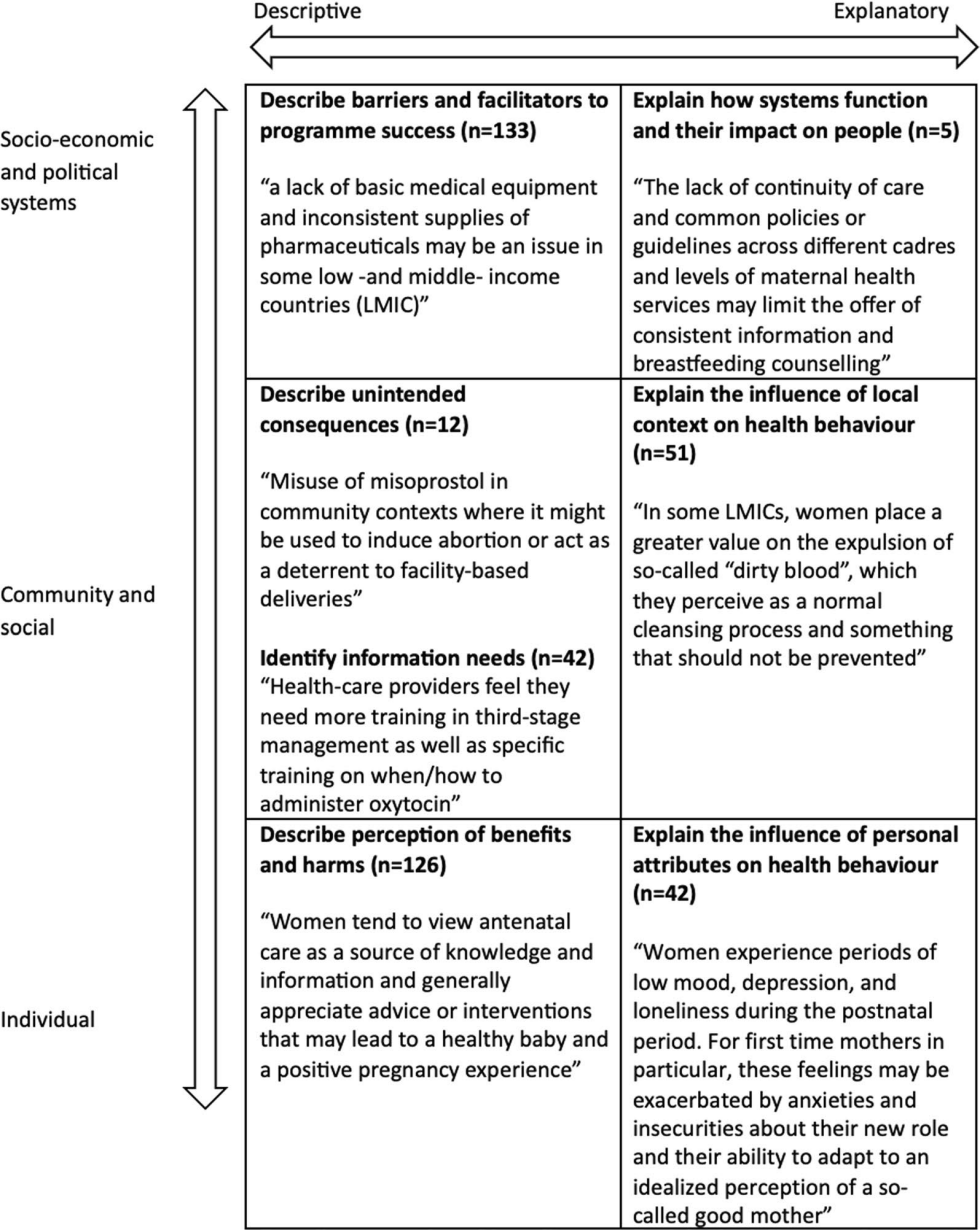
Illustration of how WHO guidelines used qualitative research according to their role

Overall, qualitative research summarised in guidelines typically provided descriptive understanding of logistical barriers and facilitators to programme success (133 quotations across 18 guidelines) or patient perception of benefits and harms (126 quotations across 18 guidelines). Less frequently, qualitative findings explained the influence of local context of health-seeking behaviours and the influence of local context (51 quotations across 18 guidelines); described information needs (42 quotations across 10 guidelines); explained the influence of personal attributes on health seeking behaviour (42 quotations across 10 guidelines); described unintended consequences (12 quotations across 5 guidelines); or explained how systems function and their impact on individuals (5 quotations across 1 guidelines). Figure 3 provides an overview of these findings with selected example quotations derived from the guidelines presented in this study. A cross comparison of how these roles fed into each stage of the decision-making process is presented below:

#### Retrieving qualitative research

Guideline documents either specifically commission research to inform their process or identify existing literature. Seven guidelines commissioned QES specifically for their guideline [22, 25, 28–32], while four guidelines performed a systematic search of published literature [19, 20, 33, 34]. However, seven guidelines did not include any methods for how they obtained qualitative research.

Overall, the guidelines in our analysis drew on a total of 38 primary studies and 25 systematic reviews of qualitative research (QES) to inform their recommendations. Guidelines most often drew on 2 qualitative research studies and a maximum of 33 qualitative research studies [27].

All guidelines that used qualitative research drew on systematic reviews of qualitative evidence, known as QES to inform their process. No guidelines drew exclusively on primary qualitative studies, but five did include them alongside qualitative evidence synthesis data [22, 24, 25, 27, 31].

#### Informing guideline scope

Seven guidelines [19–25] on the topic of MANH drew on the same QES [26] to inform the addition of a quantitative ‘positive postnatal experience outcome’, defined as ‘*in which women, partners, parents, caregivers and families receive information and reassurance in a consistent manner from motivated health workers. Both the women’s and babies’ health, social and developmental needs are recognized, within a resourced and flexible health system that respects their cultural context*’ [25]. The inclusion of this outcome allowed for prioritising women’s psychosocial and emotional well-being alongside physical health outcomes, such as mortality or morbidity, when evaluating an intervention. One infectious disease guideline [27] drew on qualitative research to inform the background of 6/99 recommendations. Findings here were often coded as ‘to understand why programmes succeed or fail’, suggesting that qualitative research can be used in this context to detail challenges with current approaches and provide a rationale for the consideration of new interventions and service designs.

#### Informing the decision to recommend an intervention

All 18 guidelines drew on qualitative research to inform the decision. The frequency of use for this purpose likely reflects the standardisation of the research-to-decision-making framework (EtD) and pre-specified domains of ‘acceptability’, ‘values and preferences’, ‘feasibility’ or ‘equity’ of a proposed intervention. Table 2 provides an overview of which of these domains included qualitative research. Regarding the feasibility of the proposed intervention, 12 guidelines drew on qualitative research. Regarding the acceptability of the proposed intervention, 13 guidelines drew on qualitative research. Regarding the values and preferences relating to the proposed intervention, 17 guidelines drew on qualitative research. Finally, regarding the equity implications of the proposed intervention, five guidelines drew on qualitative research.

Findings that described people’s perception of benefits and harms were typically used in the ‘values and preferences’ domain, which helped to understand the importance patients place on guideline outcomes. They were also used to inform acceptability and feasibility, and it was noted that typically these findings often justified that interventions were acceptable or feasible. In contrast, unacceptable or unfeasible aspects of interventions drew on findings concerned with explaining the influence of local context on health seeking behaviour, understanding how programmes succeed or fail or identifying information needs. Qualitative research was rarely used to identify unintended consequences or to understand how systems function and its impact. These two roles may have important contributions to considerations of equity, yet few guidelines drew on qualitative research to inform this domain.

Depending on the information provided, all but one [27] of the guidelines then determined a judgement of ‘probably yes’, ‘variable’ or ‘probably no’ to each domain. Judgements of the former two were frequent, and we observed only one occasion in which the acceptability was judged to be ‘probably no’ [25]. However, ‘varied’ acceptability judgements did not appear to correspond to context specific recommendations or feed into implementation considerations. We found only one example where qualitative research had influenced the overall recommendation and was directly reported in the accompanying justification [25].

#### Informing the implementation considerations of an intervention

We found only one guideline where qualitative research had been clearly cited in the designated implementation considerations section for 1/99 recommendations [27]. This makes it difficult to assess the extent to which qualitative research is used for this component or to delineate considerations that are derived from panel opinion or other forms of research. The qualitative research finding used here stated ‘other challenges include lack of nutrition support’ in reference to adherence support required for children and infants. The reductive nature of the quotation makes it difficult to assess the intended purpose of the use of qualitative research.

### Standards of qualitative research use

#### Certainty

All MANH guidelines reported judgements about the certainty of evidence by applying the CERQual tool to their QES findings but did not conduct any formal quality assessment on stand-alone primary studies. None of the infectious disease guidelines reported judgements about the certainty of evidence or conducted quality assessments on primary studies.

#### Transparency

Readers should be able to understand the justification for each recommendation from the research presented [12]. However, we found that this information was often lacking. A rationale for why the guideline panel judged there to be ‘probably yes’, ‘varied’ or ‘probably no’ acceptability, feasibility, and equity was not provided in any guideline. For some recommendations, the judgement could be easily intuited. For example, a summary of qualitative research that only describes positive viewpoints under acceptability could be reasonably judged to be ‘probably yes’. Yet, when varied viewpoints were presented, it was unclear why acceptability had been labelled ‘probably yes’ as opposed to ‘varied’. Was this due to the relative proportions of conflicting viewpoints or the relative importance of viewpoints?

Some guidelines drew on a mixture of both qualitative and quantitative studies to inform their values, acceptability, feasibility and equity domains. When this occurred, it was not clear how this research was weighed and evaluated in the decision. For example, in one guideline [27], women were less accepting of the intervention in qualitative interviews in contrast to the surveys which reported high rates of acceptability. Yet the guideline summarised acceptability as ‘high’ and cited quantitative studies to support this. As no quality assessments were performed in any guideline, it is likely that weighting was not dependant on this.

#### Specificity

Qualitative research can allow for more tailored recommendations that moves beyond what intervention may work in a controlled setting, to which intervention may work in real-life settings and contexts. This is often referred to as the efficacy to effectiveness gap [35]. However, for this to happen contextualising of recommendations are necessary. This requires narrative summaries of qualitative research to retain sufficient information on the context of findings [12]. A total of 11 guidelines contextualised a finding at least once. However, overall contextualisation was infrequent and reductive as considerations were labelled as: LMIC settings (54 findings), low-resource settings (2 findings), rural settings (7 findings), HIC settings (3 findings), children (1 finding) and unequal gender relations (2 findings). We acknowledge there is likely to be some crossover between these considerations but have listed them as referred to in the guideline documents. Moreover, contextualised findings did not appear to lead to more nuanced recommendations, e.g. for which populations is this intervention acceptable, or implementation considerations, e.g. how should the implementation be adapted for specific populations.

#### Reflexivity

Three guidelines in the field of MANH health, but no guidelines in infectious diseases, included someone experienced in qualitative research on the panel. Meanwhile, we sought to understand whether summaries of qualitative research had been produced by the guideline author team, by the guideline panel or in close collaboration, yet no guidelines reported this.

## Discussion

Qualitative research was frequently used in WHO guidelines between 2020 and 2022, although had a larger role in informing MANH than infectious diseases. Within healthcare, qualitative research has its roots in nursing, due to the relative importance of social interventions [6], and it is likely that the frequent use of qualitative research in MANH is linked to its longer history here, given the similarities in the two fields. This may also explain why some of the MANH guidelines included qualitative expertise, compared with none of the infectious disease guidelines.

We found that qualitative research rarely informed the scope of the guideline or the implementation considerations. Instead, qualitative research most often informed the decision. A similar study reported that 86% of WHO and UK, US and Canadian national guidelines used qualitative research to inform decisions but only 20% to identify clinical questions and 19% to inform implementation considerations [36]. This may be due to lack of clear citing, which made it difficult to assess accurately the extent of use. However, qualitative research presented in guidelines were often found to touch on issues regarding implementation, and yet this information did not appear to track to the appropriate section. Given that qualitative methods are considered an integral component in wider implementation science, it is surprising to see the lack of qualitative research here [37, 38].

Across the different theoretical aims of qualitative research, the most frequently used was ‘describing perception of benefits and harms’ and ‘describing barriers and facilitators to programme success’. In contrast, explanatory findings were less frequently used. Similarly, In National Institute of Health and Care Excellence (NICE) guidelines between 2015 and 2019, over half of qualitative research addressed one of two types of question: “What are the barriers and/or facilitators?” and “What are the information (and support) needs?”, and they were all descriptive in nature [39]. This may indicate a limited understanding of the potential of qualitative research particularly for more explanatory findings or simply reflect that they infrequently capture these findings to begin with. However, engaging with explanatory findings may allow guideline panels to indicate to national government which findings are likely to be transferable to their context and population groups.

We found that summaries of qualitative research and the process of transforming these into ‘yes’, ‘no’ or ‘varied’ judgments were often reductive, at the expense of the original case complexity and nuance [8, 40]. There are several ways recommendations can be contextualised from the perspectives of: geographical, epidemiological, sociocultural, socioeconomic, ethical, legal and political [41]. Qualitative research may help in understanding how proposed interventions interact with these aspects of context, but this is currently poorly conducted. One driver of this may be in how domains such as ‘acceptability’ are framed and defined. Guideline developers drew on the following definition of acceptability: ‘the extent to which that intervention is considered to be reasonable among those receiving, delivering or affected by the intervention’ [13]. However, acceptability can include affective attitude, burden, ethicality, intervention coherence, opportunity costs, perceived effectiveness and self-efficacy [42].

We found that guidelines failed to address or consider quality when interpreting primary qualitative research. Similarly, national UK guidelines by the National Institute of Health and Care Excellence (NICE) between 2003 and 2019 rarely conducted quality appraisal [39, 43]. High quality, rigorous evidence is central to the principles of evidence-based practice [1], and it is important that appropriate standards are applied to qualitative research, not only to ensure the usability of the findings but also to institutionalise the credibility of the methodology as a whole. The use of qualitative research also lacked transparency as it was often unclear how the information had been interpreted and evaluated. Aside from a transparency issue, it is possible that qualitative research was just not a key influencer in most decisions and mainly relegated to supportive roles in guideline processes [44].

### Study limitations

This study has some limitations. First, we collected guideline documents from a relatively short time frame. The trends documented in this review may be an artefact of 2020–2022, specifically, and do not describe general trends in qualitative research use. Second, lack of clear and transparent reporting on the use of qualitative research does not necessarily mean that, for example, it did not directly feed into overall judgements, or implementation considerations. Document analysis is limited by the availability of public documents, and it may be that further information is contained within meeting notes, email exchanges and other private reports that we cannot access. Finally, we chose to focus on the topics of MANH and infectious disease as they account for a large portion of qualitative research, but the use of qualitative research may be different for other topic areas.

## Conclusions

Qualitative research frequently informed WHO guideline decisions particularly in the field of MANH and was rarely used to inform guidelines relating to infectious diseases. However, the process of how qualitative evidence was used and evaluated often lacked transparency. We identified unmet potential in informing implementation considerations and contextualisation of the recommendations. Use in these areas needs further methods development.

### Supplementary Information

**Additional file 1: Excluded studies.**

## Data Availability

The coded guideline documents and data extraction tables are available from the authors upon request.

## Abbreviations

MANH: Maternal and newborn health
WHO: World Health Organisation
NICE: National Institute of Health and Care Excellence
QES: Qualitative evidence synthesis
LMIC: Low-and middle-income country
HIC: High income country
EtD: Evidence to decision
