# Supplementary material for: Use of qualitative research in World Health Organisation guidelines: a document analysis
Source: Health Res Policy Syst. 2024 Apr 4;22:44. doi: 10.1186/s12961-024-01120-y (PMC10996230; doi:10.1186/s12961-024-01120-y)
Supplement: Supplementary file 1 — Additional file 1: Excluded studies. [file 12961_2024_1120_MOESM1_ESM.docx]

## Excluded guidelines table

| Date | Title | Exclusion reason |
| --- | --- | --- |
| 5 March 2020 | Improving early childhood development. | Child health, not specific to newborn. |
| 21 April 2020 | WHO guideline on use of ferritin concentrations to assess iron status in individuals and populations. | Nutrition intervention, not specific to maternal and newborn populations. |
| 28 September 2020 | WHO guideline on country pharmaceutical pricing policies. | Pharmaceutical pricing; Not relevant. |
| 28 September 2020 | Guidelines on mental health promotive and preventive interventions for adolescents. | Adolescent health |
| 22 December 2020 | Guidelines on the management of chronic pain in children. | Child health, not specific to newborn. |
| 8 April 2021 | WHO guideline on the dairy protein content in ready-to-use therapeutic foods for treatment of uncomplicated severe acute malnutrition. | Nutrition intervention, not specific to maternal and newborn populations. |
| 22 June 2021 | WHO guideline on school health services. | Child health, not specific to newborn. |
| 22 September 2021 | WHO global air quality guidelines: particulate matter (‎PM2.5 and PM10)‎, ozone, nitrogen dioxide, sulfur dioxide and carbon monoxide. | Environment pollution; Not relevant. |
| 27 October 2021 | Guideline for clinical management of exposure to lead. | Environment pollution; Not relevant. |
